# Bevacizumab Induces Upregulation of Keratin 3 and VEGFA in Human Limbal Epithelial Cells in Vitro

**DOI:** 10.3390/jcm8111925

**Published:** 2019-11-09

**Authors:** Maria Notara, Anna Lentzsch, Thomas Clahsen, Sara Behboudifard, Gabriele Braun, Claus Cursiefen

**Affiliations:** 1 Deptement of Ophthalmology, University of Cologne, Faculty of Medicine and University Hospital Kerpener Straße 62, 50937 Cologne, Germany; anna.lentzsch@uk-koeln.de (A.L.); thomas.clahsen@uk-koeln.de (T.C.); sarabehboudi@yahoo.de (S.B.); gabriele.braun@uk-koeln.de (G.B.); claus.cursiefen@uk-koeln.de (C.C.); 2 Centre for Molecular Medicine Cologne (CMMC), University of Cologne, Robert-Koch-Straße 21, 50931 Cologne, Germany

**Keywords:** Bevacizumab, limbal epithelial cells, stem cells, keratin 3

## Abstract

Topical application of vascular endothelial growth factor A (VEGFA) inhibitors including Bevacizumab is used for antiangiogenic therapy at the ocular surface. While clinical studies have suggested that this approach is well-tolerated, the effect of the drug on limbal epithelial stem cells has not been studied. In this study, the effect of Bevacizumab on phenotype and functionality of putative limbal epithelial stem cells (SC) was investigated. The effect of Bevacizumab on human limbal epithelial cells was assessed in terms of metabolic activity and scratch wound closure. The different treatment groups featured no difference in proliferation and colony forming efficiency (CFE) of limbal epithelial cells or their putative SC marker expression. A significant delay in scratch closure of all the Bevacizumab-treated groups was detected at 4 h. RNA and protein quantification indicated a dose-responsive increase of keratin 3. VEGFA RNA expression also increased while VEGFC and D as well as VEGFR1, 2 and 3 were unchanged. This study highlights previously unknown effects of Bevacizumab on cultured putative limbal epithelial SC: a dose-related increase of keratin 3, an increase in VEGFA as well as a delay in scratch wound closure. These in vitro data should be considered when using Bevacizumab in the context of limbal epithelial SC transplantation.

## 1. Introduction

Corneal avascularity and transparency are essential for normal vision. Absence of corneal blood and lymphatic vessels is mediated by a delicate network of inhibitory mechanisms in the cornea [1]. An intact corneal epithelium is necessary for transparency and refraction [2]. This epithelial layer is constantly replenished by a limbal epithelial stem cell (LESC) population residing in the basal epithelial layer of the limbus [3,4]. Dysfunction or depletion of limbal epithelial cells (limbal stem cell deficiency, LSCD) results in persistent corneal inflammation, corneal surface conjunctivalization, corneal neovascularization and recurrent epithelial defects [5,6]. Conditions leading to LSCD and therefore neovascularization are chemical and thermal burns, inflammatory eye diseases, persistent hypoxia (contact lens wear) [7] as well as genetic disorders such as aniridia [8]. Patients with LSCD suffer from photophobia, reduced visual acuity and pain due to recurrent ocular surface defects. In severe cases, LSCD can result to blindness [4].

Vascular endothelial growth factor (VEGF) is considered to be one of the key mediators of angiogenesis. VEGF belongs to a platelet-derived growth factor family and includes several isoforms such as VEGFA, VEGFB, VEGFC, VEGFD, VEGFE and placental growth factor (PlGF) while their actions are mediated via their receptors, namely VEGFR1, VEGFR2 and VEGFR3 [9,10]. The cornea maintains its avascularity with a dynamic balance of proangiogenic and anti-angiogenic signals. For example, the ectopic corneal epithelial expression of sVEGFR1, sVEGFR2 and epithelial VEGFR3 function as “sinks” to VEGFA, C and D thus preventing corneal (lymph)angiogenesis [2,11,12]. VEGF promotes several steps of angiogenesis including immune cell recruitment, proteolytic activities, as well as proliferation, migration and capillary tube formation of endothelial cells [10,13]. As the upregulation of VEGF is a key factor in corneal neovascularization, a targeted therapy with anti-VEGF antibodies seems to be a promising symptomatic treatment option [14,15].

The off label use of topical antiangiogenic agents is an attractive treatment option for corneal neovascularization. Drugs including Ranibizumab (Lucentis^®^), an anti-VEGF antibody [1], and Aflibercept (Eylea^®^), an anti-angiogenic compound recognizing ligands of VEGF receptors 1 and 2 [2], have been reported to have beneficial antiangiogenic effects following topical application in vascularized corneas [3,4]. Bevacizumab (Avastin^®^) is a whole humanized monoclonal anti-VEGF antibody that binds all isoforms of VEGFA and neutralizes their activity [16]. In recent years, the off-label use of Bevacizumab via intravitreal injection has revolutionized the treatment of various retinal vascular diseases [17]. Further studies evaluated the beneficial effects of anti-VEGF agents on treating anterior segment disease [1,14,15]. Bevacizumab is considered to have no cytotoxic effects on human corneal epithelial and fibroblast cells [18]. Animal models and clinical trials confirmed the antiangiogenic efficacy of Bevacizumab by either subconjunctival injection or by topical application to reduce corneal neovascularization in various conditions. [14,15,19,20]. In vitro evidence of the antiangiogenic effect of the drug has also been reported [5]. While it is generally well tolerated by patients, it was found that a small percentage may develop new corneal epithelial defects as a result of the treatment [6]. Further studies are needed to establish dosage safety and methods of administration [21].

Since topical anti-VEGFs have been used in the context of limbal stem cell transplantation [14,15,22,23] and since larger trials on limbal epithelial stem cell therapy such as Holoclar^®^ are underway [24], the effect of anti-VEGFs on epithelial stem cells becomes relevant. The specific effect of Bevacizumab on limbal epithelial stem cell phenotype has not yet been investigated. The aim of this study was to evaluate the effect of Bevacizumab on the limbal epithelial stem cell functionality and phenotype, including putative stem cell marker expression, colony forming efficiency as well as its effect on the expression of the proangiogenic factors VEGFA, C and D as well as their respective receptors VEGFR1, 2 and 3.

## 2. Materials and Methods

### 2.1. Culturing of 3T3 Mouse Fibroblasts

A 3T3 mouse fibroblast cell line was grown in Dulbecco’s Modified Eagle Medium (DMEM, Life Technologies, Darmstadt, Germany) with added 10% Foetal Bovine Serum (Gibco, Darmstadt, Germany) and 1% penicillin/streptomycin/amphotericin (Life Technologies, Darmstadt, Germany). The culture medium was exchanged every other day and the cells were sub-cultured upon reaching 60%–70% confluence at a ratio of 1:10. The cultures were maintained at 37 °C and at 5% CO_2_ in air. For use as a feeder layer for the propagation of corneal epithelial cells, the 3T3 fibroblasts were growth-arrested in a culture medium containing 6 μg/mL mitomycin C (Sigma, Munich, Germany) for a period of 3 h.

### 2.2. Primary Human Limbal Epithelial Cell Harvesting and Maintenance

Human corneo-scleral rims were donated for research purposes and following ethics approval, a surplus of surgery, were used for cell isolation in accordance with the Declaration of Helsinki (University of Cologne local ethics committee, approval number 15–093). Human limbal epithelial (HLE) cells were grown in a medium containing DMEM-F12 (1:1) (Life Technologies, Darmstadt, Germany) with added 10% Fetal Bovine Serum, 1% penicillin/streptomycin/amphotericin (Life Technologies, Darmstadt, Germany), 5μg/mL human recombinant insulin (Sigma, Munich, Germany), 0.1 nM cholera toxin B (Sigma, Munich, Germany), 0.05 mM hydrocortisone (Sigma, Munich, Germany) and 10 ng/mL epidermal growth factor (Life Technologies, Darmstadt, Germany). The culture medium was exchanged every other day. HLE cells were isolated from corneas provided by the Cornea Bank of the Department of Ophthalmology, University of Cologne, Germany. The tissue was treated with a 1.2 U/mL dispase II solution (Sigma, Munich, Germany) for 2 h at 37 °C or overnight at 4 °C. After the treatment, it was transferred into a 10 cm petri dish. The epithelial cells were gently scraped by using a feathered scalpel aiming at the limbal border to achieve an enriched LESC/progenitor population. The isolated cells, (approximately 3 x 10^5^), were collected by using 5 mL epithelial culture medium and then placed into a T-25 tissue culture flask (Nunc, Schwerte, Germany) containing a feeder layer of growth-arrested 3T3 fibroblasts at a cell density of 2.4 × 10^4^ cells/cm^2^. The cultures were kept at 37 °C and 5% CO_2_ in a humidified incubator.

### 2.3. Treatment with Bevacizumab

HLE cells were treated with four concentrations of Bevacizumab: 0.125 mg/mL, 0.25 mg/mL, 0.50 mg/mL and 1 mg/mL for different times depending onto each assay as described in the sections below. These concentrations were chosen based on previous reports studying in vitro effects of Bevacizumab on corneal epithelial cells and fibroblasts [7,8]. These concentrations were prepared using a stock solution of 25 mg/mL Bevacizumab in serum-free DMEM/F12 supplemented with 0.1% BSA. For the proliferation, scratch wound and colony forming efficiency assay, the drug substrate (sub) as described in the Bevacizumab leaflet, was used as a control in the equivalent amounts corresponding to the four different Bevacizumab concentrations (respectively referred to as sub1–4). The substrate consisted of 240 mg trehalose dihydrate, 23.2 mg sodium phosphate (monobasic, monohydrate), 4.8 mg sodium phosphate (dibasic, anhydrous) and 1.6 mg polysorbate 20 (all from Sigma, Munich, Germany) in 4 mL of DDH_2_O. The selection of Bevacizumab concentrations and controls was based on previous reports [25,26].

### 2.4. Immunocytofluorescence

In order to identify changes in the phenotype of limbal epithelial cells following different treatments a panel of markers associated with different levels of cellular differentiation within the limbal epithelium has been used, namely integrin β1, p63α and keratin 3. Integrin β1, a marker normally observed in the basal and some of the suprabasal limbal epithelium [27] and the transcription factor p63α [28] are associated with limbal stem and progenitor cells and are absent in the superficial layers where the epithelial cells are considered terminally differentiated. ABCG2 and N-cadherin are also associated with the putative limbal stem cell phenotype [9,10]. Keratin 3, on the other hand, is only expressed in the superficial layers of the cornea and is completely absent in basal and suprabasal cells [29].

The cells were plated in CNT-57 serum-free media (CELLnTEC Advanced Cell Systems, Bern, Switzerland) 8 well permanox chambered slides (labtek, Nunc, Schwerte, Germany) and after 24 h they were exposed to the different treatment groups. After 5 days, the cells were washed three times with PBS, fixed for 10 min at room temperature in 4% (wt/vol) paraformaldehyde. The samples were blocked for 1 h in PBS supplemented with 5% goat serum (Sigma, Munich, Germany) and 0.5% Triton X (Sigma, Munich, Germany) followed by the rabbit polyclonal integrin beta 1 antibody from Abcam (Cambridge, UK), mouse monoclonal antibody for keratin 3 from Millipore (Darmstadt, Germany) and rabbit polyclonal antibody for P63α from New England Biolabs (Frankfurt am Main, Germany) or blocking reagent only (negative control) overnight at 4 °C. Due to the lack of a commercially available antibody of the ΔΝP63α isomer, which is more specific to putative stem cells [28], an antibody recognizing both the ΔΝ and the TA variants was used instead and may therefore mark both stem cells and transient amplifying cells.

Subsequently, the cells were incubated with their respective secondary antibody (goat anti-rabbit alexa 488 and goat anti-mouse alexa 647, both from Life Technologies, Darmstadt, Germany), washed and counterstained with DAPI (Sigma, Munich, Germany). All incubations except the primary antibody incubation were performed at room temperature, and each step was intermittent with 3 × 5 min rinses with PBS supplemented with 0.1% tween-20 (Sigma, Munich, Germany). Negative controls were treated in the same way, except for omitting the primary antibody step. A minimum of 3 random fields of each immunostained sample were photographed by using a Zeiss LSM880 confocal microscope and representative images are presented in figures. Immunofluorescence was repeated with cells from at least 3 different donors.

### 2.5. Colony-Forming Efficiency Assay

Limbal epithelial cells were plated in 6 well plates at a density of 5 × 10^5^ cells per well and left to settle for 24 h in CNT-57 media as described in the previous section. Subsequently, they were treated with Bevacizumab or with the controls. The cells were grown in these conditions for 5 days and then the colony-forming efficiency (CFE) assay was performed.

For CFE assay [30,31], 3T3 fibroblasts were used as a feeder layer. The cells were growth arrested with mitomycin C (Sigma, Munich, Germany) as in the section above and plated at a cell density of 4.8 × 10^5^ cells in each well of a 6-well plate. HLE were seeded at a clonal cell density of 1000 cells per well of the 6-well plate. On day 12, the cultures were fixed with cold methanol for 20 min at −20 °C. Subsequently, the colonies were stained with a solution of 1% rhodamine B (Sigma, Munich, Germany) and 1% Toluidine Blue (Sigma, Munich, Germany) for 30 min at 37 °C. Finally, the plates were photographed and Image J software was used to count the number of colonies that measured greater than 2 mm diameter. The percentage of colony-forming efficiency was calculated by using the Equation (1):

(1) $$\mathrm{CFE}\;\left(\%\right)=\frac{\mathrm{Number}\;\mathrm{of}\;\mathrm{colonies}>2\mathrm{mm}}{\mathrm{Number}\;\mathrm{of}\;\mathrm{cells}\;\mathrm{seeded}}x\;100$$

The experiments were carried out with cells from a minimum of three different donors (*n* = 6).

### 2.6. Cell Metabolic Activity

The metabolic activity was evaluated by using the Alamar Blue (AB) assay (Thermo Scientific, Schwerte, Germany). Limbal epithelial cells were seeded in 96 well plates in CNT-57 media and at a cell density of 5 × 10^3^ cells per well in a minimum of 8 replicates. The next day, the culture medium was replaced with the different Bevacizumab treatment groups. The assay was carried out after 24 h.

To perform the assay, the cultures were incubated for 3 h in 100 μL/well alamar blue reagent diluted 10 times in PBS (with *n* = 8 at minimum). Cell-free wells with added alamarBlue reagent were used as blanks. After the incubation, the plates were measured in an Epoch plate reader (BioTek, Bad Friedrichshall, Germany) in absorbance mode at 570 nm and 600 nm and the percentage of reduction of the alamar blue reagent was calculated as recommended in the manufacturer’s instructions. These experiments were repeated with cells from a minimum of 3 different donors.

### 2.7. Scratch Wound Assay

Limbal epithelial cells were plated to complete confluence in a 96 well plate and serum-starved for 2 h. Subsequently the scratches were made in each well by using a 10 μL pipette tip (*n* = 8). Then, the cells were treated with the various Bevacizumab concentrations and controls. The wounds were photographed at 0, 4 and 16 h. The wound surface areas at each time-point were measured using Image J software. The data of each replicate were calculated as a percentage of the healed scratch area compared to the original scratch area at 0 h. The experiments were performed a minimum of five times with cells from a minimum of 5 different donors.

### 2.8. RT-PCR

Limbal epithelial cells were plated on T25 flasks at a seeding density of 7 × 10^5^ cells/flask and were left overnight to adhere. The following day, the cells were exposed to the various treatment groups over a period of 5 days. The culture media was replaced every 48 h in order to replenish the Bevacizumab. Messenger RNA was isolated by using an RNeasy Micro Kit (Qiagen, Valencia, CA, USA). A minimum of 25 ng cDNA, 0.4 μM corresponding forward and reverse primer and SsoFast EvaGreen Supermix (Bio-Rad, Hercules, CA, USA) were used per RT-PCR reaction. The primers were designed using Primer BLAST (Basic Local Alignment Search Tool, National Centre for Biotechnology Information) and their sequences are displayed on Table 1. The TATA Box Binding Protein (TBP) was used as the housekeeping gene. For each RT-PCR reaction an initial denaturation step of 95 °C for 2 min was followed by 40 cycles at 95 °C for 5 s and at 56 °C for 15 s. Finally, a denaturation step for 60 s at 95 °C was added. All PCR reactions were performed in triplicate and a non-template control (NTC) was included in all experiments. Experiments were repeated three times with cells from three different donors.

### 2.9. Western Blotting

Sub-confluent cell monolayers of each cell treatment group were cultured for 5 days, then lysed on ice with RIPA buffer (Sigma), followed by centrifugation of the lysates at 12.000 rpm for 10 min and storage of the supernatants at −85 °C until use. Protein extracts (1.5 mg/mL) were separated at 10% SDS-PAGE gel and transferred at a polyvinylidene fluoride (PVDF) membrane. Immunoblotting was performed using the same β1 integrin, P63α and keratin 3 antibodies used for immunostaining, an anti-rabbit β-actin antibody (housekeeping protein) from New England Biolabs (Frankfurt am Main, Germany) and a mouse or rabbit secondary antibody coupled to horseradish peroxidase (DAKO, Waldbronn, Germany). The membranes were developed using an enhanced chemiluminescence reagent (Bio-Rad, Munich, Germany) and the images were captured using a Bio-Rad Molecular Imager^®^ Gel Doc™ XR System. Semi-quantification was carried out using image J. Signals from each group was normalized against beta actin and the control group (0 mg/mL Bevacizumab) was set as 1.

### 2.10. Statistical Analysis

Statistical analysis of results was done by using the GraphPad Prism 6.05 software (GraphPad, San Diego, CA, USA). One-way Analysis of Variance (ANOVA) with Tukey’s multiple comparisons test was used to analyze the data. Sets of data producing a *p* value lower than 0.05 were considered statistically significant. The experiments were performed using a minimum of 3 experimental triplicates and repeated at least three times (using cells from a minimum of three different donors to reflect biological variability). All error bars displayed in graphs represent standard deviation of the mean.

## 3. Results

### 3.1. Bevacizumab Does Not Affect Limbal Epithelial Cell Proliferation While It Decelerates Scratch Wound Healing

**Figure 1:**
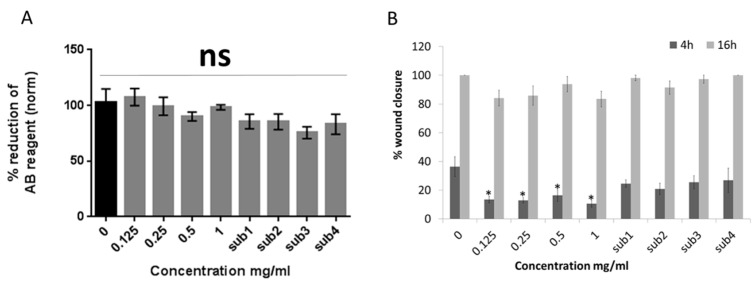
Bevacizumab does not affect the metabolic activity albeit hinders the scratch wound healing of human limbal epithelial cells. alamarBlue (AB) assay results at 24 h after treatment, indicate that there is no statistically significant effect of different Bevacizumab concentrations on limbal epithelial cell metabolic activity (**A**). On the other hand, scratch closure was significantly delayed for all Bevacizumab concentrations at 4 h but not at 16 h timepoint. (**B**). The drug substrate (sub) was used as control in the equivalent amounts corresponding to the four different Bevacizumab concentrations (respectively referred to as sub1–4) For both assays, number of replicates *n* = 8 and * *p* < 0.05. Error bars represent standard deviation of the mean.

Firstly, the effect of Bevacizumab on the metabolic activity of human limbal epithelial cells was investigated by using alamar blue (AB) assay 24 h after the beginning of the treatment (Figure 1A). The results demonstrated that there was no significant effect of any drug concentration (0.125–1 mg/mL) or the equivalent concentrations of the drug substrate (sub1–4) compared to the control (0 mg/mL Bevacizumab, no substrate).

Conversely, a scratch wound assay exhibited that all Bevacizumab concentrations caused a delay in scratch wound closure of HLE cells at a 4 h time-point (*p* < 0.05 for all Bevacizumab concentrations compared to the control). At a 16 h time-point, there was a similar trend however, this was not significant. The drug substrate had no effect on the percentage of scratch wound closure in either time-point (Figure 1B). Representative scratch wound assay microphotographs of the different treatment groups are featured in Supplementary Figure S1

### 3.2. Colony Forming Efficiency (CFE) and Putative Limbal Epithelial Stem Cell Marker Expression Remains Unaffected by Bevacizumab While the Differentiation Marker Keratin 3 is Upregulated

In order to investigate whether Bevacizumab has an effect on limbal epithelial stem cell phenotype, a colony forming efficiency assay as well as an analysis of the markers β1 integrin p63α and keratin 3 was performed by using immunofluorescence RT-PCR and Western blotting.

**Figure 2:**
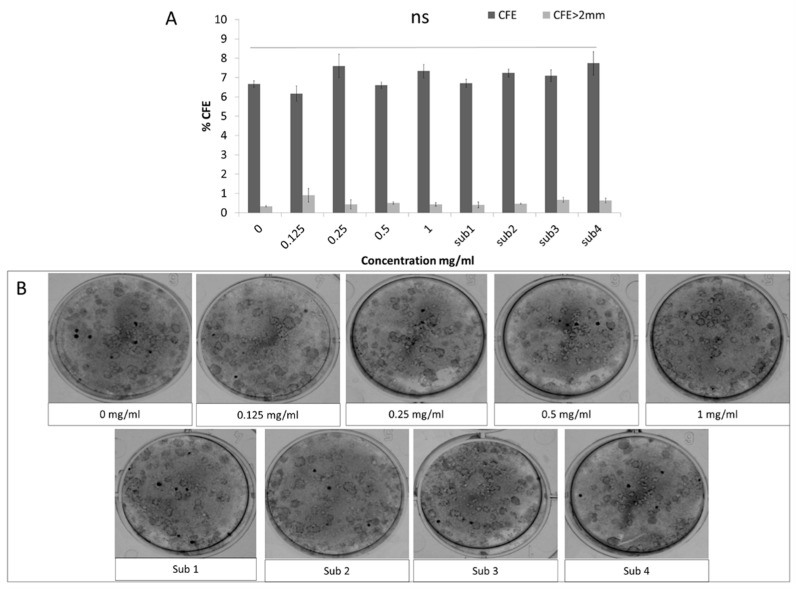
The colony forming efficiency (CFE) of presumed limbal epithelial stem cells remained unaffected following Bevacizumab treatment. Total colonies percentage as well colonies with a diameter greater than 2 mm were unaffected by Bevacizumab treatment (**A**). Panel B shows representative Rhodamine B colony stainings from all treatment groups (**B**). Number of replicates *n* = 6.

Specifically, 5 days after treatment there was no significant effect of any of the Bevacizumab concentrations on the total number of colonies or the ones with a diameter larger than 2 mm (Figure 2A, representative colonies of all treatment groups depicted in Figure 2B).

**Figure 3:**
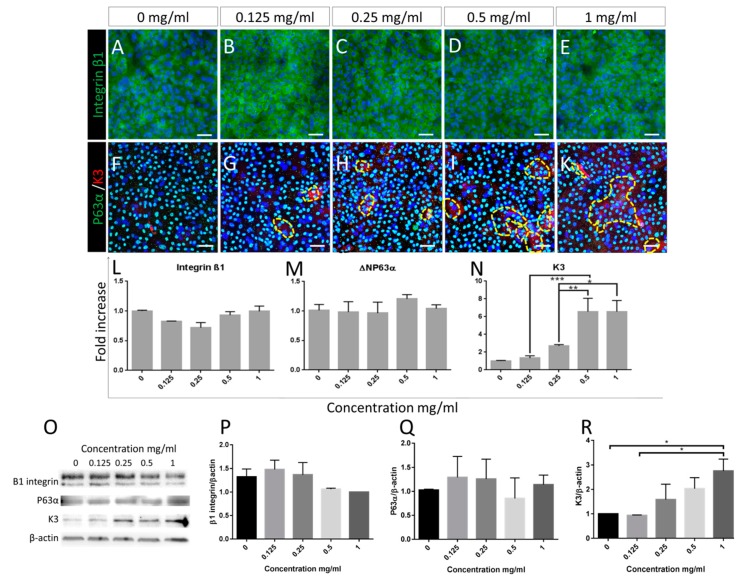
Putative limbal epithelial stem cell marker expression is not affected by Bevacizumab, except Keratin 3 levels which increase following treatment. Immunofluorescence staining of the basal marker integrin β1 (**A–E** alexa488) and of p63α (**F**–**K** alexa488) as well as RNA and protein expression (densitometry analysis of Western blotting) of integrin β1 and ΔΝΡ63α (**L**,**M** and **P**,**Q** respectively) remained unchanged following treatment with Bevacizumab. In contrast, the mature corneal epithelial marker keratin 3, localised in cells with flattened and enlarged morphology (**F–K**, alexa555, positive areas surrounded by yellow dotted lines) exhibited a significant and dose responsive increase following treatment with Bevacizumab as shown by both QPCR assessment (**N**) and Western blotting densitometry analysis (**R**). Representative blots are depicted in panel (**O**). For immunofluorescence: number of replicates *n* ≥ 3. Scale bars correspond to 50 μm. For RT-PCR and Western blotting, number of replicates *n* = 3 and * *p* < 0.05, ** *p* < 0.01, *** *p* < 0.001. In densitometry analysis (**P–R**), control group set as 1.

Similarly, as demonstrated by immunocytofluorescence data also following 5 days of treatment, no significant difference between treatment groups was observed in the expression of β1 integrin and p63α (Figure 3A–E and Figure 3F–G, respectively, Alexa 488). These data were backed up by RNA and protein expression analysis by RT-PCR and Western blotting (Figure 3L,O,P for integrin β1 and Figure 3M,O,Q for p63α). Similarly, no change was observed in the expression of the putative stem cell markers ABCG2 and N-Cadherin (Supplementary Figure S2).

In contrast, immunofluorescence data indicated an increase of keratin 3-positive cells following a 5-day treatment with Bevacizumab (surrounded by yellow dotted lines, Figure 3F–K, Alexa 555). RT-PCR, as well as Western blotting analysis, confirmed these data exhibiting a dose-responsive significant upregulation of keratin 3 RNA and protein ranging up to 8-fold and 3-fold respective increase compared to the control (Figure 3N,O,R respectively, * *p* < 0.05, ** *p* < 0.01, *** *p* < 0.001).

### 3.3. VEGFA Expression of Limbal Epithelial Cells Is Upregulated in Response to Treatment with Bevacizumab

**Figure 4:**
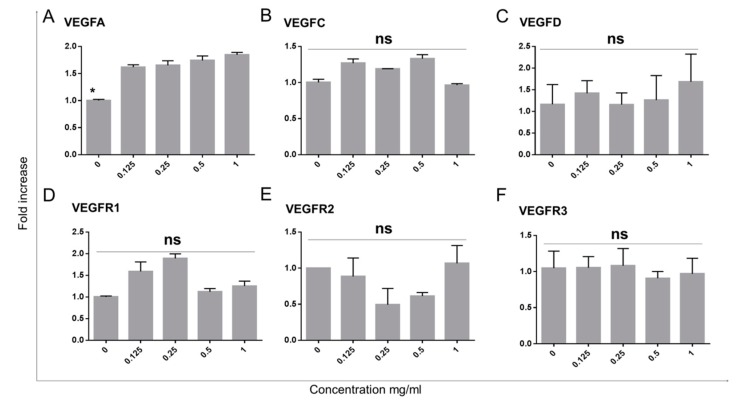
VEGFA expression of limbal epithelial cells is increased with Bevacizumab treatment while VEGFC, D and VEGFR1, 2 and 3 remain unchanged. VEGFA RNA expression exhibited a small (1.5-fold) but significant increase following Bevacizumab treatments (**A**). VEGFC and D were unchanged (**B**,**C**). No differences were observed in the expression of VEGFR1, 2 and 3 (**D–F**). Number of replicates *n* = 3 and * *p* < 0.05.

In order to assess the response of limbal epithelial cells to Bevacizumab in terms of molecules regulating (lymph)angiogenesis, we have assessed the RNA expression of VEGFA, VEGFC and VEGFD as well as their respective receptors VEGFR1, VEGFR2 and VEGFR3. The RT-PCR data indicated that VEGFA featured a small (1.5-fold) but significant upregulation (*p* < 0.05 for all Bevacizumab concentration ranging from 0.125–1 mg/mL) of RNA expressed in all treatment groups in relation to the control (Figure 4A).

In contrast, the RNA levels of VEGFC-D as well as VEGFR1-3 did not feature statistically significant changes in response to treatment with any of the Bevacizumab concentrations (Figure 4B–C and Figure 4D–F respectively).

## 4. Discussion

Our study allows for the following conclusions to be drawn: (a) Bevacizumab does not affect putative corneal limbal epithelial stem cell proliferation and CFE. In contrast, scratch wound healing is significantly delayed. (b). While stem cell marker expression is unaffected by Bevacizumab, differentiation markers such as Keratin 3 and proangiogenic factors such as VEGFA are upregulated in response to Bevacizumab. (c) This suggests cautious use of Bevacizumab in the context of limbal epithelial stem cell transplantation in patients.

The metabolic activity data revealed that there was no reduction of epithelial cell proliferation following Bevacizumab treatment for any of the concentrations used. As the primary function of Bevacizumab is the neutralization of VEGF, it is expected to have an inhibitory effect on the proliferation of endothelial cells especially on vascular endothelial cells as the action of VEGFA via its receptors VEGFR1 and 2 is the master regulator of their metabolic activity [9]. In previous in vivo [32] and in vitro studies, it was demonstrated that while Bevacizumab had no cytotoxic effect on various ocular cell types including choroidal endothelial cells, retinal pigment epithelial cells of human and rat origin [33,34], it delayed, as expected the proliferation of human choroidal endothelial cells [34]. Regarding corneal endothelial cells, while Bevacizumab causes no toxicity, a significant dose dependent reduction of their proliferation is observed [35]. While there are conflicting reports linking Bevacizumab cytotoxicity to corneal fibroblasts [18,36], it was shown that there were neither cytotoxic nor anti-proliferative effects on an immortalized human corneal epithelial cell line [18] while histological assessment showed no significant impact in mouse corneal epithelium [32]. Our data confirm the latter observation in primary human limbal epithelial cells.

Even though Bevacizumab is considered a non-toxic and effective treatment of corneal neovascularization various groups have observed that, in both animal models and in the clinical practice, prolonged use of Bevacizumab is linked to delayed corneal epithelial wound healing, spontaneous epitheliopathy and stromal thinning [37,38,39,40]. Our scratch wound assay data confirmed that Bevacizumab impeded scratch closure of limbal epithelial cell cultures. This may be attributed to changes in the cells adhesion properties: previous reports using an immortalized corneal epithelial cell line illustrated that Bevacizumab induced downregulation of integrin α and β, which was confirmed by both RNA and protein measurements; therefore it interfered with the adhesion mechanisms that are essential for cell migration [41].

In addition to proliferation and wound healing assessment, we have investigated the effect of Bevacizumab on the putative limbal epithelial stem cell phenotype. It was found that the colony forming efficiency as well as the levels of P63a [28] which is associated with putative limbal stem cells and transient amplifying cells, remained unaffected by Bevacizumab. The basal epithelial marker β1 integrin [27] was also unchanged. However, a dose responsive increase of the corneal epithelial differentiation marker keratin 3 was observed following Bevacizumab treatment. In the human cornea, the superficial epithelial layers produce the highest levels of keratin 3 [29]. Although it has already been demonstrated by histology that cells located in these superficial layers of the corneal epithelium produce lower levels of VEGF [42], it has so far not been reported that the VEGF blockade leads to the upregulation of keratin 3 thus driving epithelial cell differentiation. This keratin 3 increase appears incompatible with the concurrent stable expression of the putative stem cell markers and unchanged CFE. A possible explanation is that the culture conditions favor a basal phenotype and only a small number of cells express keratin 3; therefore, while the change in keratin 3 is detected any changes in the other markers and CFE are not significant. The exact effects of VEGF blockade on corneal epithelial differentiation dynamics are not as yet explored and merit investigation as the subject in a separate study.

RT-PCR analysis indicated that Bevacizumab caused a small but significant increase in RNA levels of VEGFA, while VEGFC and D, as well as VEGFR1, 2 and 3, were unchanged. The minor increase of VEGFA RNA expression in response to Bevacizumab treatment can be attributed to a defense mechanism of the cells while trying to maintain stable levels of the protein as the antibody targets specifically this VEGF family member [16].

Overall, our data shed more light into the specific effects of Bevacizumab on putative limbal epithelial stem cells functionality and phenotype and contribute novel information directly relevant to its off-label clinical use as treatment against corneal neovascularization, e.g., also in the context of limbal stem cell transplantation [1]. While it was confirmed that Bevacizumab has no toxic effects on limbal epithelial cells, hindering of wound healing was observed, concurring with clinical observations where long term use of Bevacizumab caused delayed wound healing and epitheliopathies. The previously undetected Bevacizumab-induced increase in keratin 3 levels of limbal epithelial cells in vitro should be taken into consideration when using the drug as prophylaxis following engraftment of ex vivo expanded limbal epithelial stem cells [22,43] as an increase in keratin 3 positive cells could have an effect on the graft outcome. Furthermore, these data showcase a possible role of VEGF to corneal epithelial differentiation which, to date, has not been investigated [2].

## 5. Conclusions

This study on the in vitro effects of Bevacizumab on putative limbal epithelial stem cells shows that Bevacizumab treatment causes a delay in scratch wound closure. Also, Bevacizumab induced a dose-responsive increase of keratin 3 RNA and protein expression in cultured putative limbal epithelial stem cells indicating a shift towards a more differentiated phenotype. The observed increase in VEGFA RNA levels may reflect an autocrine defense mechanism of the limbal epithelial cells as they attempt to compensate for the neutralization effect of Bevacizumab. Taken together the effect of Bevacizumab on epithelial cell differentiation, migration and VEGFA expression should caution its use in the context of limbal stem cell transplantation and warrants future in vivo studies.

## Figures and Tables

**Table 1:** Primer Sequences used for RT-PCR analysis.

Gene	Sense Primer	Antisense Primer
*Keratin 3*	GGCAGAGATCGAGGGTGTC	GTCATCCTTCGCCTGCTGTAG
*ΔNP63α*	GGAAAACAATGCCCAGACTC(ΔN)	ATGATGAACAGCCCAACCTC(α-termini)
*Integrin β1*	AGTGAATGGGAACAACGAGGTC	CAATTCCAGCAACCACACCA
*VEGFA*	ACAGGTACAGGGATGAGGACAC	AAGCAGGTGAGAGTAAGCGAAG
*VEGFC*	GCCTGTGAATGTACAGAAAGTCC	AATATGAAGGGACACAACGACAC
*VEGFD*	CCGCCATCCATACTCAATTATC	CCATAGCATGTCAATAGGACAGAG
*VEGFR1*	CTACCACTCCCTTGAACACGA	GGTCCACTCCTTACACGACAA
*VEGFR2*	ACCTCACCTGTTTCCTGTATGG	GACTGATTCCTGCTGTGTTGTC
*VEGFR3*	CTCAAAGTCTCTCACGAACACG	GGTACATGCCAACGACACAG
*TBP (housekeeping)*	GTTGGTGGGTGAGCACAAG	AGGAGCCAAGAGTGAAGAACAG

## Supplementary Materials

The following are available online at https://www.mdpi.com/2077-0383/8/11/1925/s1, Figure S1: Representative microphotographs of the scratch wound assay featuring cultured limbal epithelial cells treated with the control media (sup4) 0.5 mg/mL and 1 mg/mL Bevacizumab at 0 h, 4 h and 16 h. The scratched areas are highlighted with yellow dotted lines; Figure S2: ABCG2 and N-Cadherin expression is not affected by Bevacizumab. Immunofluorescence staining of the putative limbal stem cell marker ABCG2 (A–E alexa555) and of N-Cadherin (F–J alexa488) and respective quantification of marker-positive cells (K, L). Both markers remained unchanged following treatment with Bevacizumab.
