# Supplementary figures and images for: Bevacizumab Induces Upregulation of Keratin 3 and VEGFA in Human Limbal Epithelial Cells in Vitro
Source: J Clin Med. 2019 Nov 9;8(11):1925. doi: 10.3390/jcm8111925 (PMC6912829; doi:10.3390/jcm8111925)

0h

4h

16h

sup4

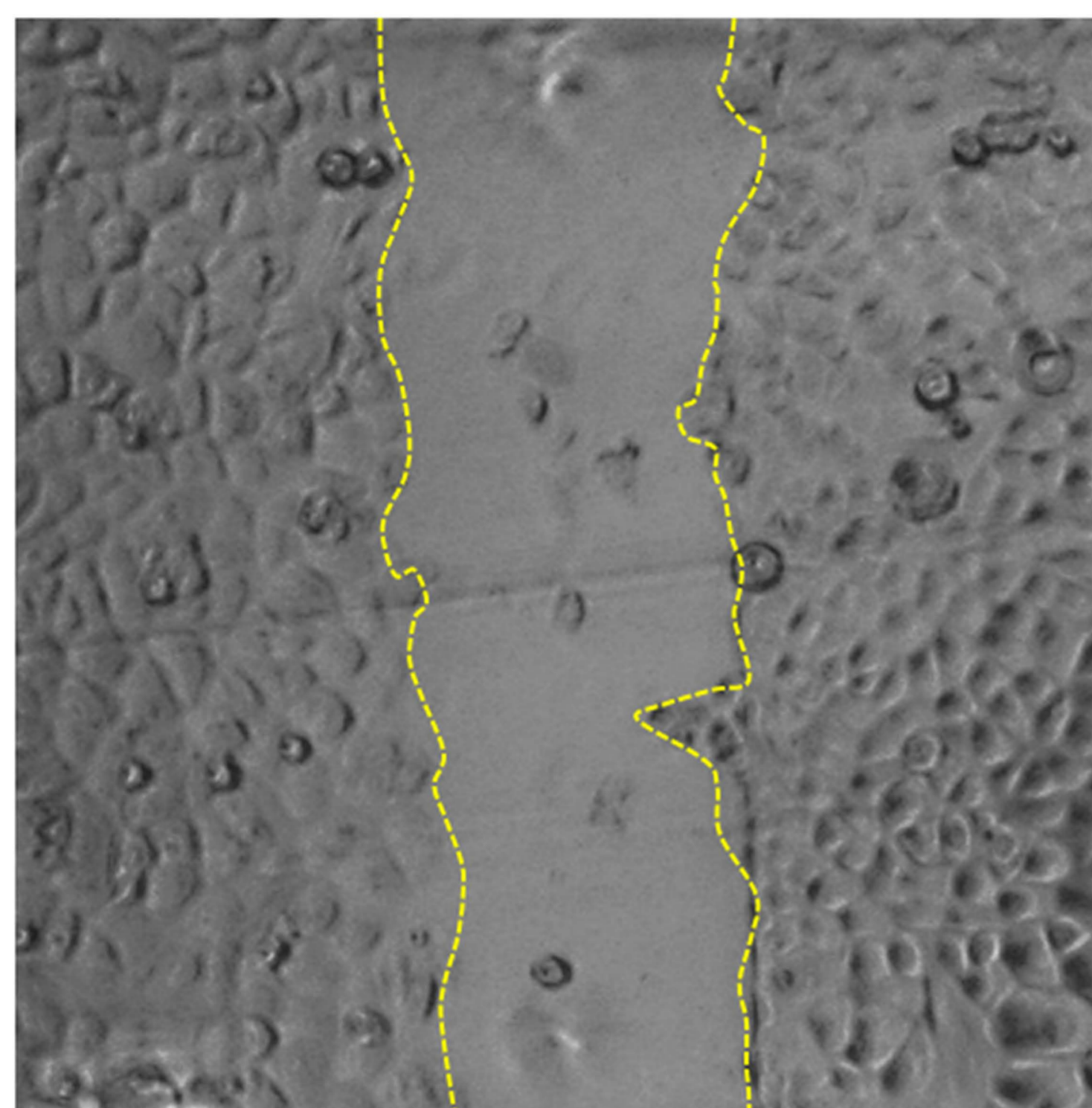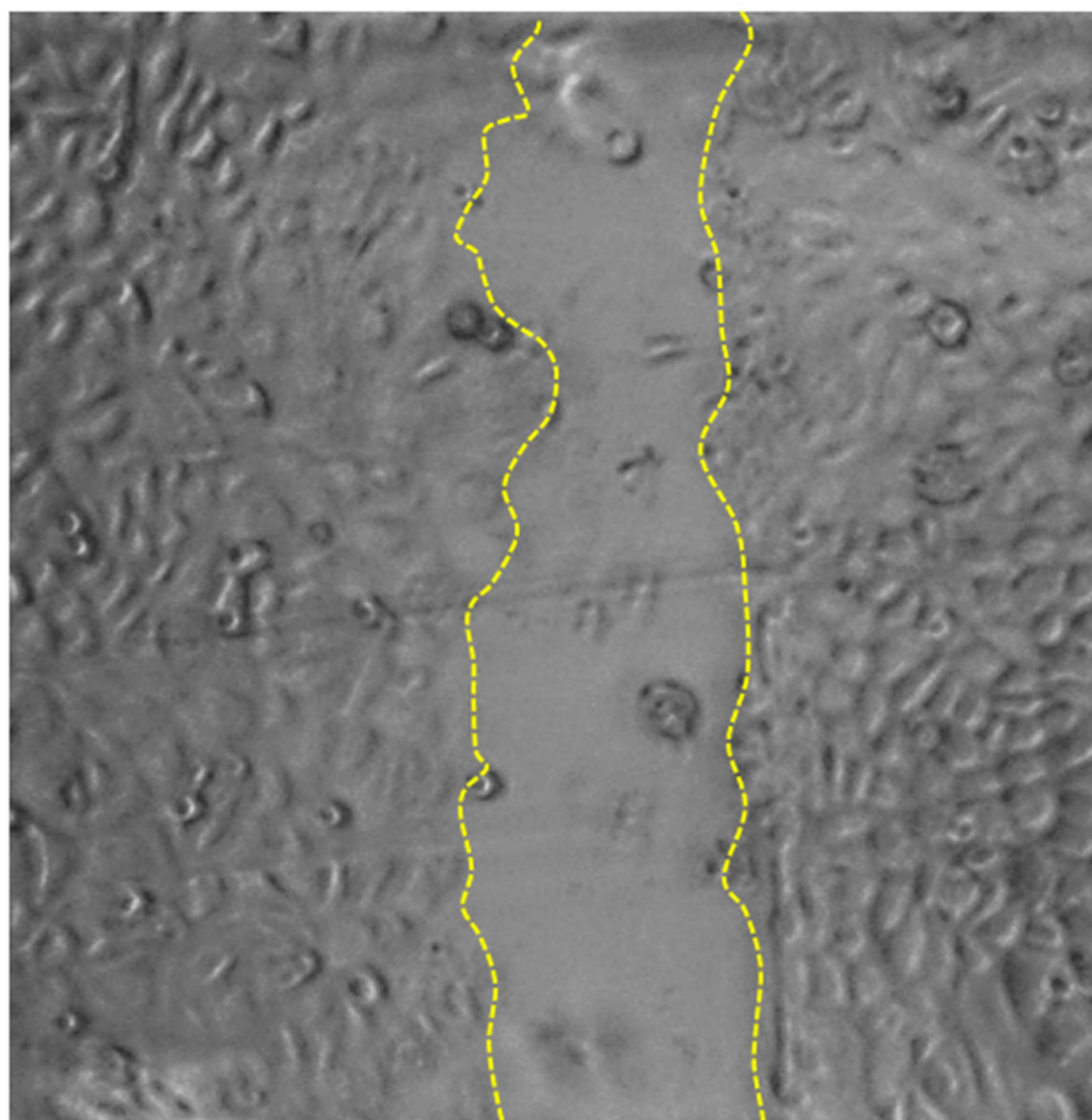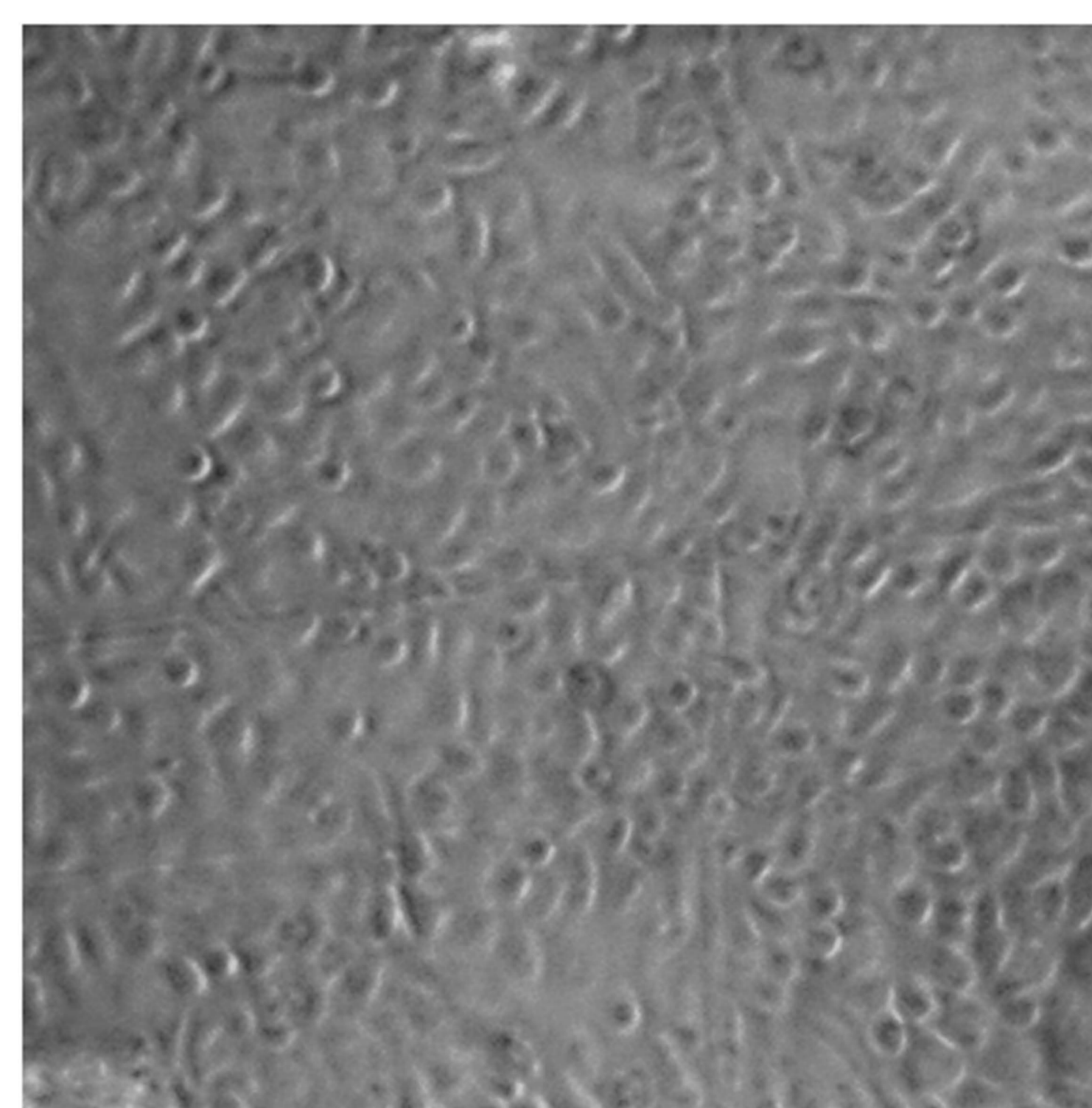

0.5mg/ml

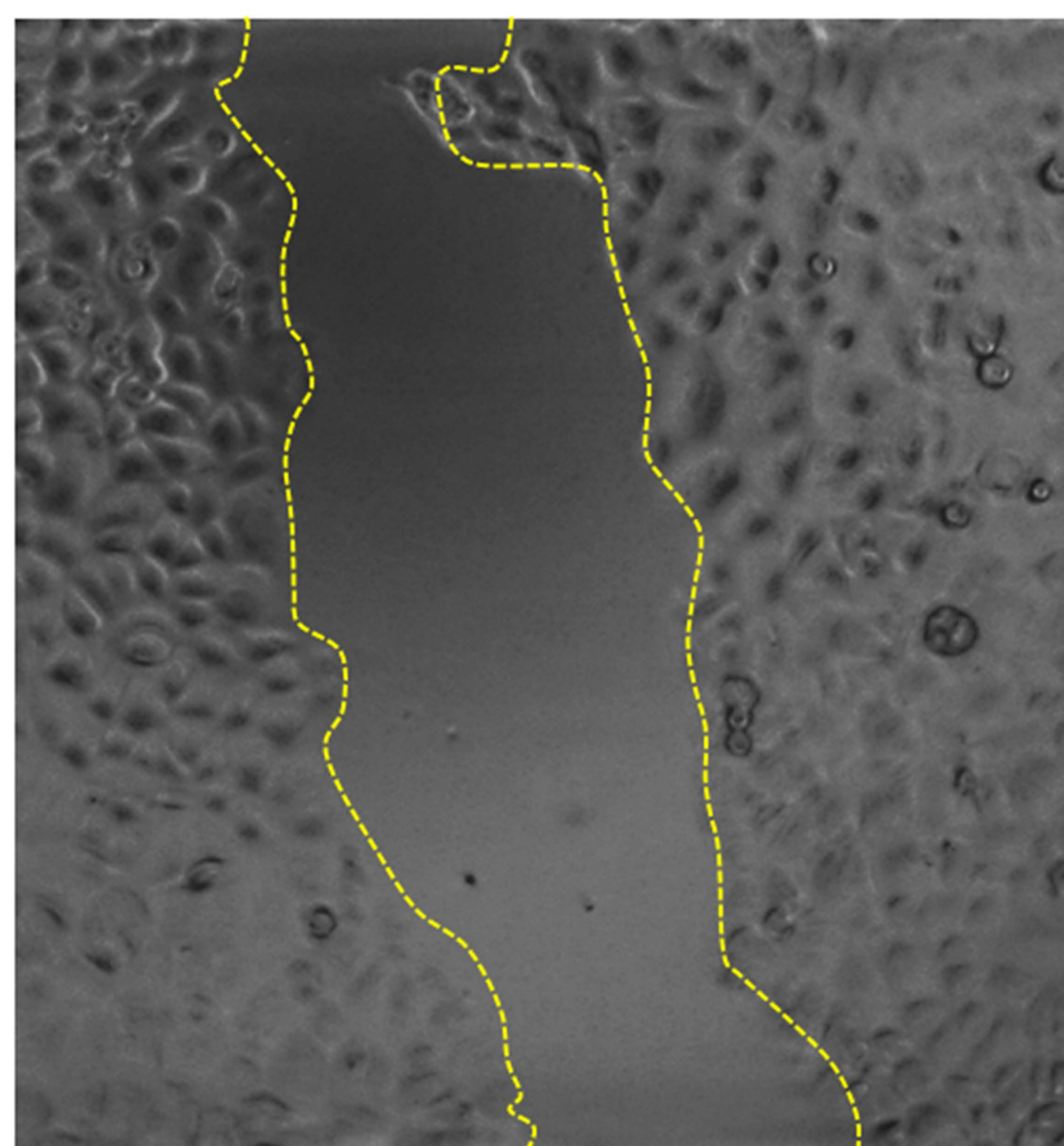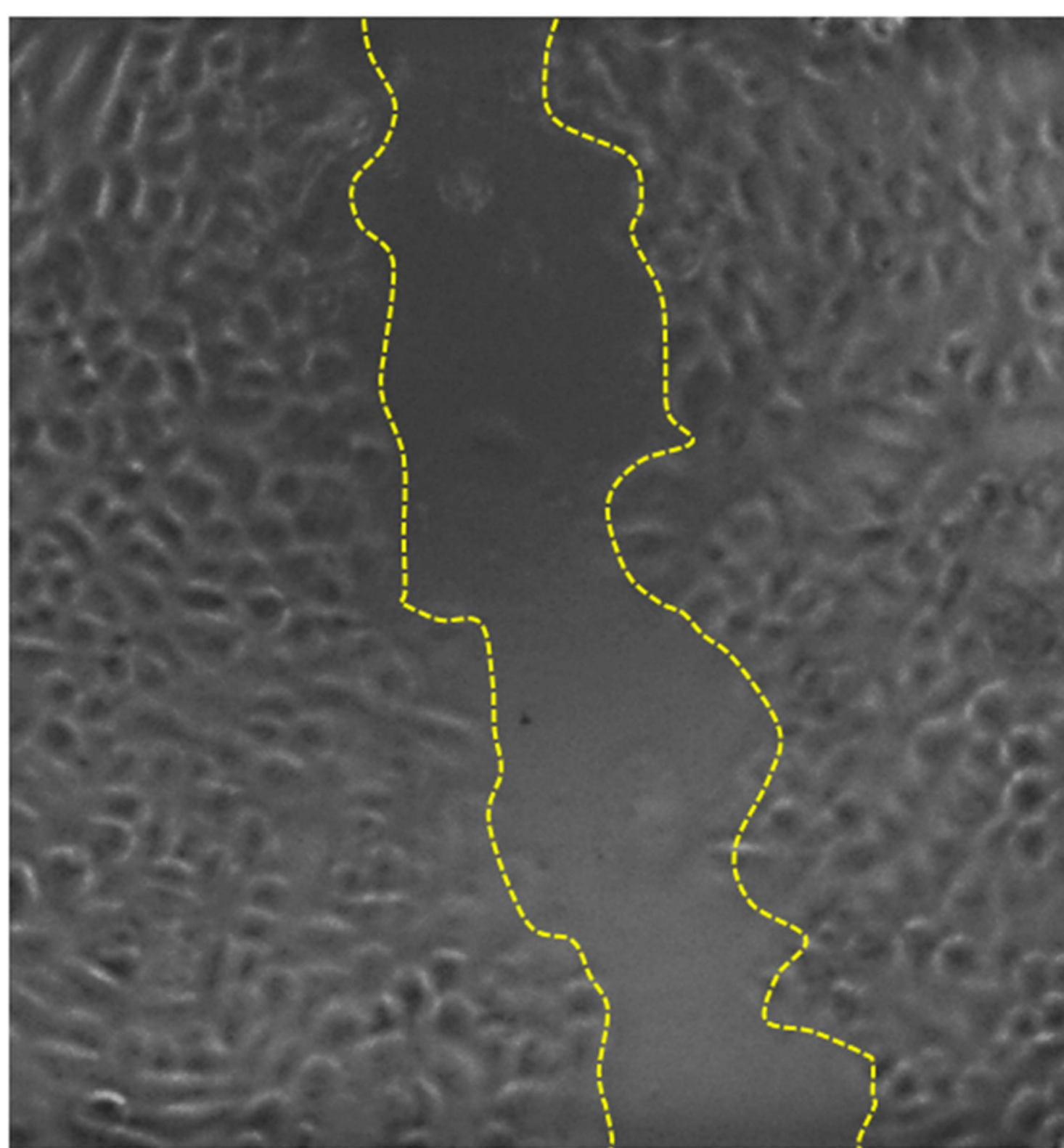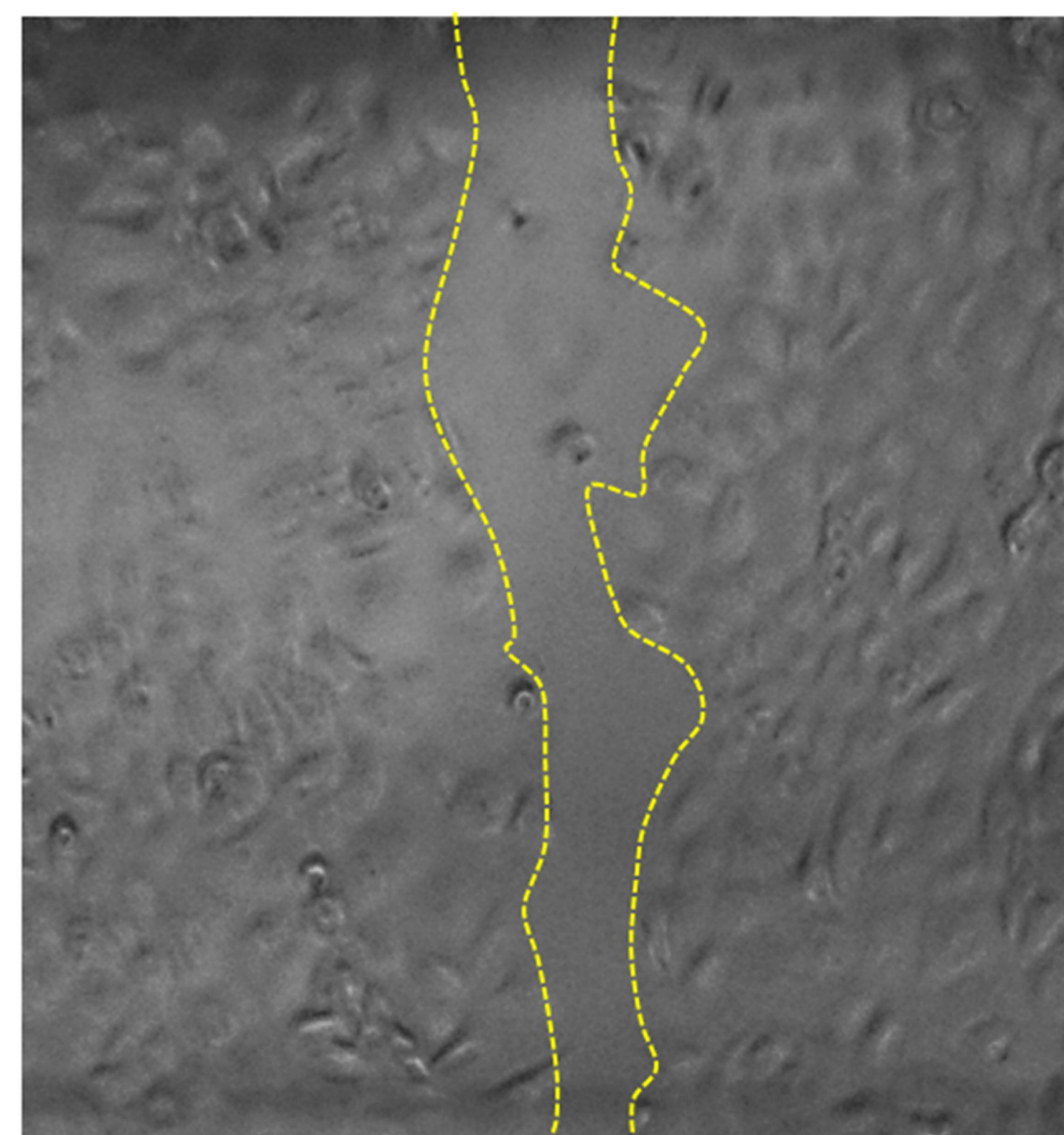

1mg/ml

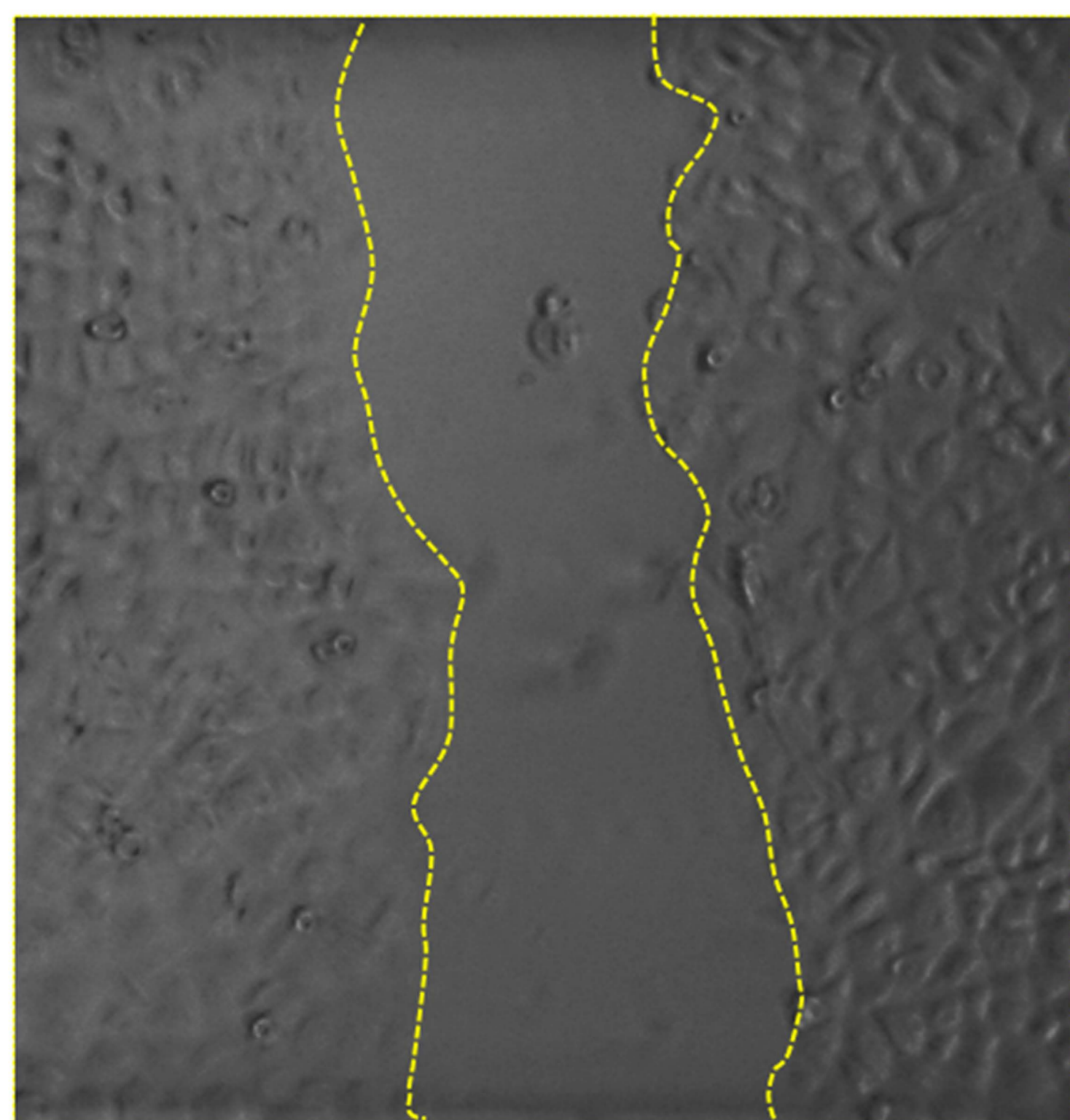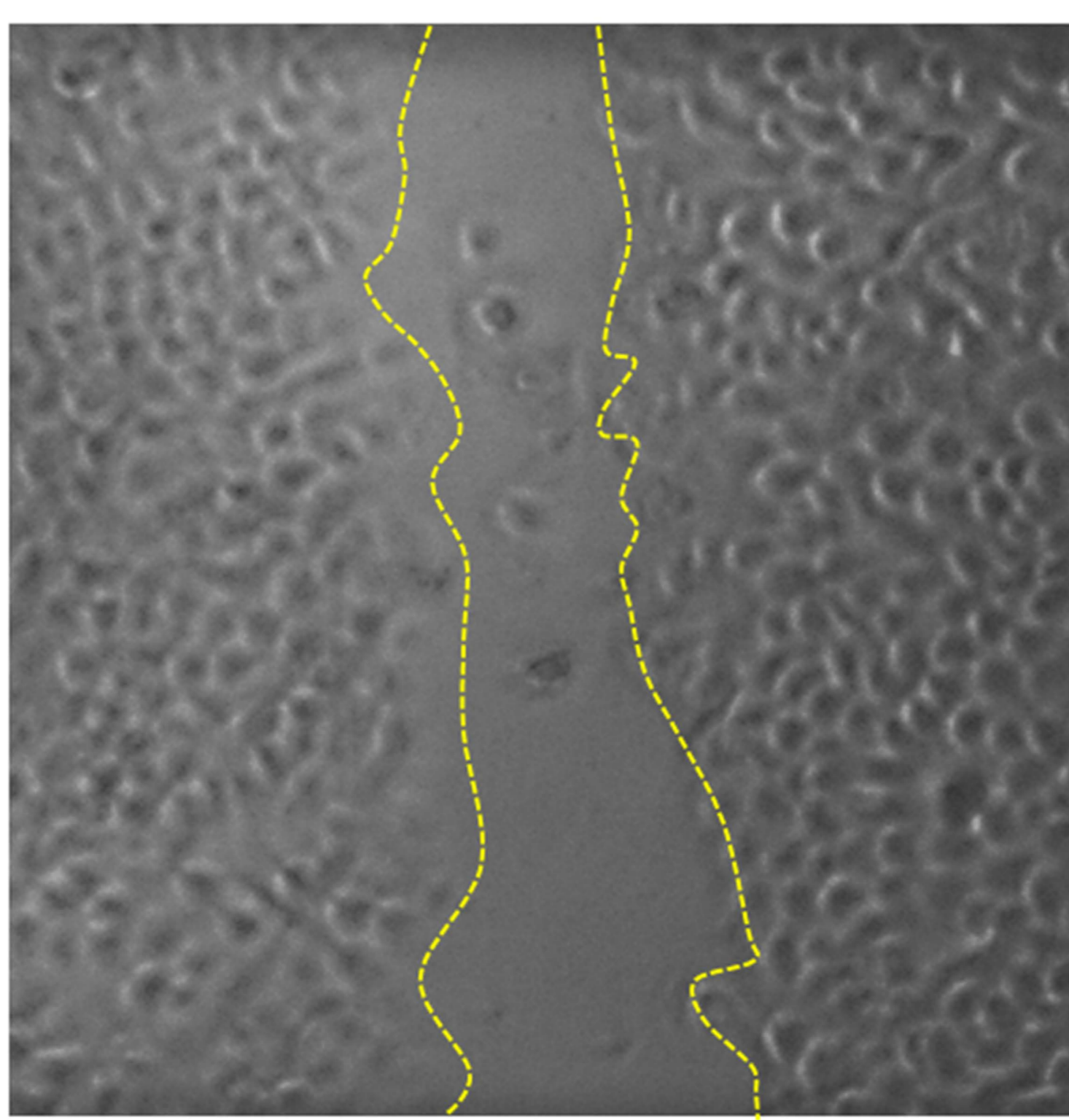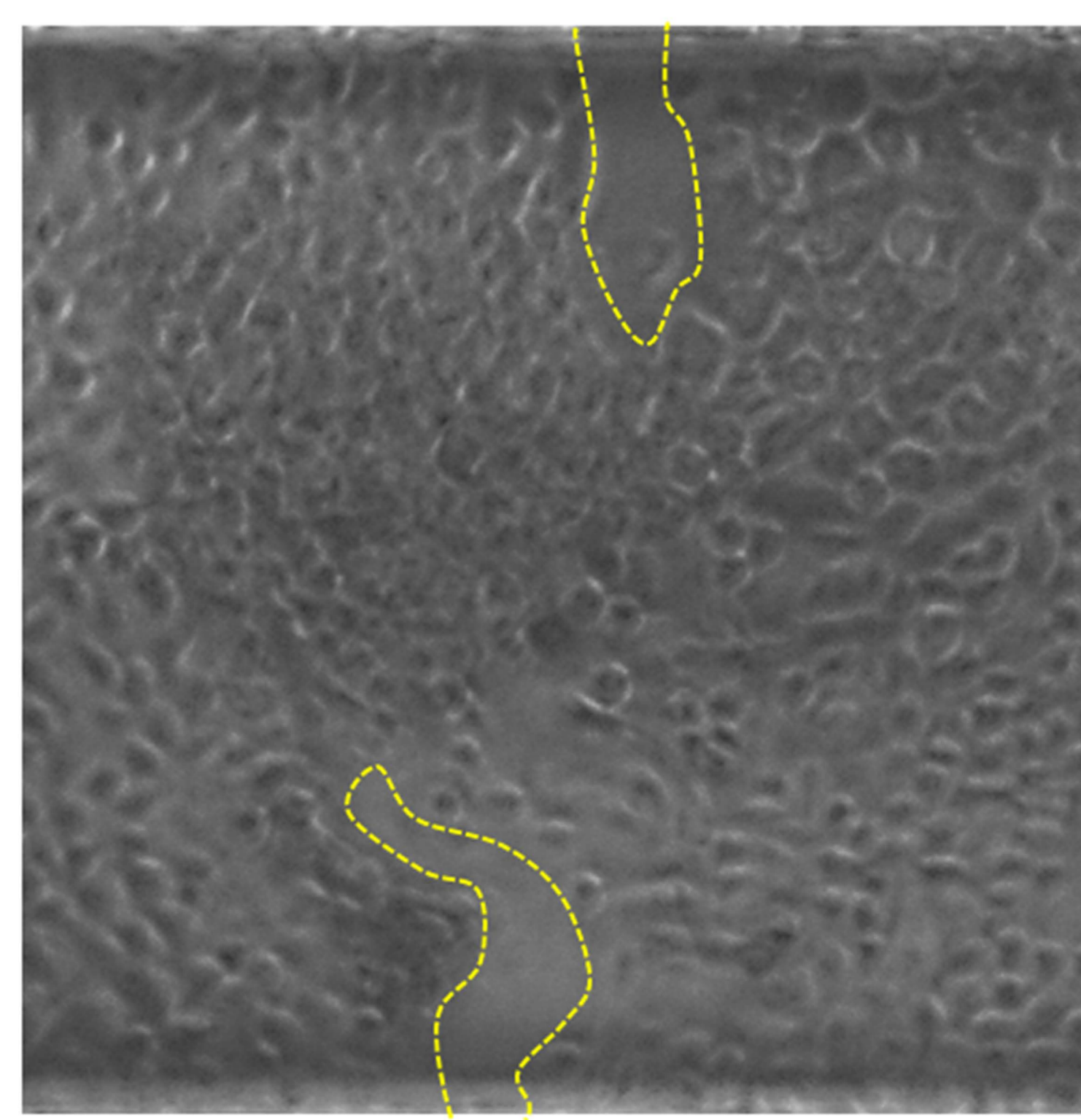

Supplement: Supplementary file 1 [file jcm-08-01925-s001.pdf]
